# Study methodology and insights from the palovarotene clinical development program in fibrodysplasia ossificans progressiva

**DOI:** 10.1186/s12874-023-02080-7

**Published:** 2023-11-13

**Authors:** Robert J. Pignolo, Mona Al Mukaddam, Geneviève Baujat, Matthew A. Brown, Carmen De Cunto, Edward C. Hsiao, Richard Keen, Kim-Hanh Le Quan Sang, Donna R. Grogan, Rose Marino, Andrew R. Strahs, Frederick S. Kaplan

**Affiliations:** 1 Department of Medicine, Mayo Clinic, Rochester, MN US; 2 Departments of Orthopedic Surgery & Medicine, The Center for Research in FOP and Related Disorders, Perelman School of Medicine, University of Pennsylvania, Philadelphia, PA US; 3 Département de Génétique, Institut Imagine, Hôpital Universitaire Necker-Enfants Malades, Université Paris Cité, Paris, France; 4 Faculty of Life Sciences and Medicine, King’s College London, and Genomics England Ltd, London, UK; 5 Pediatric Rheumatology Section, Department of Pediatrics, Hospital Italiano de Buenos Aires, Buenos Aires, Argentina; 6 Division of Endocrinology and Metabolism, the UCSF Metabolic Bone Clinic, the Eli and Edythe Broad Institute for Regeneration Medicine, and the Institute of Human Genetics, Department of Medicine, and the UCSF Program in Craniofacial Biology, University of California-San Francisco, San Francisco, CA US; 7 Centre for Metabolic Bone Disease, Royal National Orthopaedic Hospital, Stanmore, UK; 8 Ipsen, Cambridge, MA, US

**Keywords:** Fibrodysplasia ossificans progressiva, Heterotopic ossification, Clinical trial, Rare disease, Methodology

## Abstract

**Background:**

The design of clinical trials in rare diseases is often complicated by a lack of real-world translational knowledge. Fibrodysplasia ossificans progressiva (FOP) is an ultra-rare genetic disorder characterized by skeletal malformations and progressive heterotopic ossification (HO). Palovarotene is a selective retinoic acid receptor gamma agonist. Here, we describe the methodology of three studies in the palovarotene clinical development program in FOP and discuss insights that could inform future research, including endpoint suitability and the impact of trial design.

**Methods:**

PVO-1A-001 (NCT02322255) was a prospective, protocol-specified, longitudinal FOP natural history study (NHS). PVO-1A-201 (NCT02190747) was a randomized, double-blind, placebo-controlled phase II trial; PVO-1A-202 (NCT02279095) was its open-label extension. Trial designs, including treatment regimens and imaging assessments, were refined between PVO-1A-201 and PVO-‍1A-202, and within PVO-1A-202, based on emerging data as the studies progressed. Palovarotene doses were administered using a flare-up treatment regimen (higher dose for 2/4 weeks, followed by lower dose for 4/≥8 weeks; from flare-up onset), with or without accompanying chronic (daily) treatment. Flare-up and disease progression outcomes were assessed, including incidence and volume of new HO during flare-ups and/or annually, as well as other clinical, patient-reported, and exploratory outcomes. Safety was monitored throughout all studies.

**Results:**

Overall, 114 and 58 individuals with FOP were enrolled in the NHS and phase II trials, respectively. Results of the NHS and PVO-1A-201 were published in 2022; complete results of PVO-1A-202 will be publicly available in due course. Together the studies yielded important information on endpoint suitability, including that low-dose whole-body computed tomography was the optimum imaging modality for assessing HO progression annually and that long study durations are needed to detect substantial changes in functional and patient-reported outcomes.

**Conclusions:**

A flexible clinical development program is necessary for underexplored rare diseases to overcome the many challenges faced. Here, the NHS provided a longitudinal evaluation of FOP progression and interventional trials were based on emerging data. The studies described informed the design and endpoints implemented in the phase III MOVE trial (NCT03312634) and provide a foundation for future clinical trial development.

**Trial registration:**

NCT02322255 (registered 23/12/2014); NCT02190747 (registered 15/07/2014); NCT02279095 (registered 30/10/2014).

**Supplementary Information:**

The online version contains supplementary material available at 10.1186/s12874-023-02080-7.

## Background

Conducting clinical trials in rare diseases presents several challenges including small numbers of eligible participants, limited ability to conduct subsequent trials, heterogeneity across patient groups, understudied disease evolution, and a lack of validated measurement tools. Consequently, conventional trial designs are not always appropriate [1, 2].

Fibrodysplasia ossificans progressiva (FOP; OMIM #135100) is an ultra-rare, genetic disorder with an estimated global prevalence of 0.04–1.43 per million individuals [3–7]. Individuals with FOP experience sporadic and unpredictable episodes of soft tissue swelling referred to as flare-ups, which can lead to heterotopic ossification (HO) [8, 9]. In HO, the replacement of skeletal muscles and soft connective tissues with ribbons, sheets, and plates of heterotopic bone leads to immobility and compromises the cardiorespiratory system [9, 10]. As a result, individuals with FOP are usually confined to a wheelchair by the third decade of life, with life expectancy reduced to an estimated median of 56 years [9, 10]; however, progression over time is highly variable among patients [11].

FOP is caused by a spontaneous missense mutation in the Activin-like kinase 2/Activin A receptor type I (*ALK2/ACVR1*) gene, which encodes a receptor in the bone morphogenetic protein (BMP) signaling pathway [12]. Almost all individuals with FOP (approximately 97%) carry the same specific *ALK2/ACVR1* gene mutation (c.617G > A, p.R206H) [12–14]. In the presence of *ALK2/ACVR1*^R206H^, BMP signaling is enhanced, promoting chondrogenesis and HO [15, 16]. Until recently, there were no licensed treatments to prevent the formation of heterotopic bone in FOP; therapeutic approaches are generally limited to symptom management and flare-up prevention [11].

Palovarotene is an orally bioavailable, selective retinoic acid receptor gamma (RARγ) agonist [17]. RARγ agonists promote the degradation of Smad1/5/8 proteins involved in the BMP signaling pathway that are believed to be activated by *ALK2/ACVR1*^R206H^, resulting in downregulation of BMP signaling in pre-chondrogenic cells [18, 19]. Palovarotene has been shown to prevent both trauma-induced and spontaneous HO in animal models of FOP [17, 20].

**Fig. 1:**
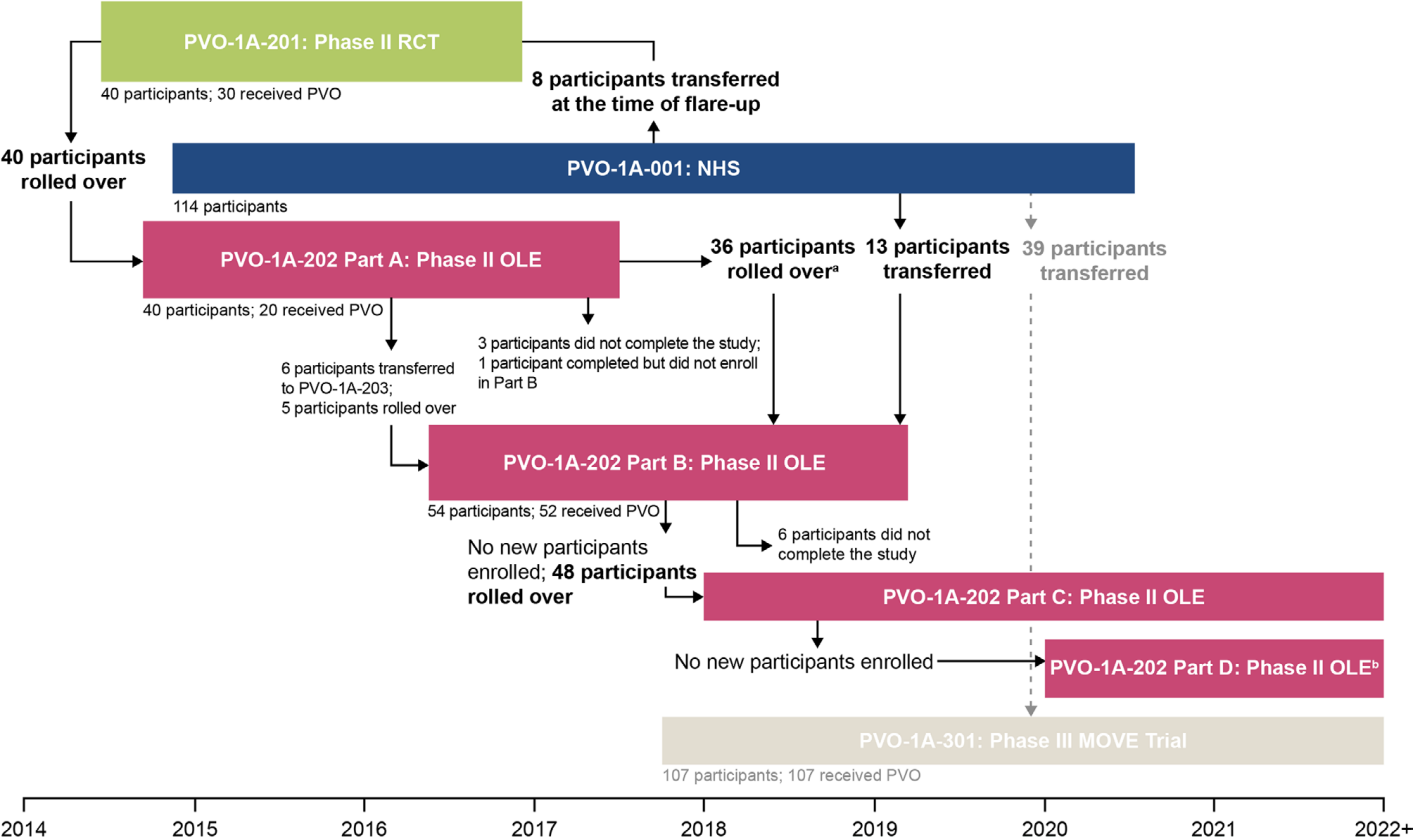
An overview of the palovarotene clinical development program for FOP. Across the clinical development program, 164 individuals with FOP had received at least one dose of palovarotene. ^a^Includes 5 new individuals who had not participated in any previous study. ^b^In PVO-1A-202 Part D annual assessments were obtained following the last dose of palovarotene in participants who were skeletally immature at the time of treatment discontinuation. FOP: fibrodysplasia ossificans progressiva; NHS: natural history study; OLE: open-label extension; PVO: palovarotene; RCT: randomized controlled trial

Clinical development programs in rare diseases are often lengthy and complex, requiring flexible and pragmatic study designs that can be modified based on new knowledge from non-clinical to phase II and III clinical trials [21, 22]. Here, we address three objectives. First, we describe the methodology of three studies included in the palovarotene clinical development program in FOP: a prospective, protocol-specified, longitudinal natural history study (NHS; PVO-1A-001 [NCT02322255; registered 23/12/2014]) [23, 24]; a randomized, double-blind, placebo-controlled phase II trial (PVO-1A-201 [NCT02190747; registered 15/07/2014]) [25, 26]; and its associated open-label extension (PVO-1A-202 [NCT02279095; registered 30/10/2014]; Fig. 1) [27]. The second objective is to discuss the challenges of designing and conducting trials in underexplored rare diseases, particularly when endpoint measures may not be clearly defined. The final objective is to outline the key lessons learned from this program that could inform future research, including the suitability of specific endpoints and how the design of a study can impact trial results, especially in an ultra-rare disease.

## Methods

### Study designs and eligibility criteria

#### PVO-1A-001 (NHS)

PVO-1A-001 (NCT02322255) was a prospective, longitudinal, 3-year, global, non-interventional NHS to evaluate, at Baseline and annually, the disease characteristics and natural progression of FOP, as well as the impact of flare-ups [24]. The key disease progression outcome was the change from Baseline in the total body burden of HO as assessed by low-dose whole-body computed tomography (WBCT; excluding the head) over 36 months. The key flare-up outcome was the incidence and volume of new HO at Week 12 as assessed by flare-up body region computed tomography (CT) scan (the full study design and schedule of assessments can be found in **Supplementary Fig. 1A**).

The NHS was conducted between December 2014 and April 2020. Individuals aged ≤ 65 years with clinically diagnosed FOP due to the *ALK2/ACVR1*^R206H^ mutation (or believed to be due to the *ALK2/ACVR1*^R206H^ mutation) who had not participated in an interventional clinical research trial within the 4 weeks prior to enrolment were eligible for inclusion. Eligible participants experiencing a flare-up during the NHS were able to enroll in PVO-1A-201. Skeletally mature participants in the NHS and all PVO-1A-202 Part A participants who had at least two symptomatic flare-ups in the past 2 years, but not in the 4 weeks prior to enrollment, with a Cumulative Analogue Joint Involvement Scale (CAJIS) score of 6–‍16 (measured on a 0–30 scale) [28], were eligible to participate in PVO-1A-202 Part B.

#### PVO-1A-201 (randomized placebo-controlled trial)

PVO-1A-201 (NCT02190747; **Supplementary Fig. 1B**) was a randomized, double-blind, placebo-controlled, multicenter phase II trial that evaluated the effect of palovarotene flare-up treatment on new HO at Week 6 and Week 12 following treatment initiation (defined as Flare-Up Day 1), which was started within 7 days of flare-up onset [26]. Investigators, participants, contract research organizations, and the central imaging laboratory were blinded. In Cohort 1, participants aged ≥ 15 years were randomized 3:1 to palovarotene 10 mg (high-dose palovarotene treatment) for 2 weeks followed by 5 mg (low-dose palovarotene treatment) for 4 weeks (palovarotene 10/5 mg flare-up treatment), or placebo for 6 weeks, followed by a 6-‍week follow-up period. In Cohort 2, participants aged ≥ 6 years were randomized 3:3:2 to palovarotene 10/5 mg flare-up treatment, palovarotene 5 mg (high-dose palovarotene treatment) for 2 weeks followed by 2.5 mg (low-dose palovarotene treatment) for 4 weeks (palovarotene 5/2.5 mg flare-up treatment), or placebo for 6 weeks, followed by a 6-week follow-up period. The primary outcome was the percentage of participants with no or minimal new HO (as defined by a HO score ≤ 3 on an analog scale from 0–6; lower score indicates less HO [29]) at the flare-up body region by plain radiograph at Week 6 (Day 42). However, given the insensitivity of plain radiographs over this timeframe, additional analyses focused on the incidence and volume of new HO at Week 12 as assessed by flare-up body region CT scan.

PVO-1A-201 was conducted between July 2014 and May 2016. Individuals ≥ 6 years old with clinically diagnosed FOP with the *ALK2/ACVR1*^R206H^ mutation, experiencing an active flare-up with ≥ 2 symptoms (see *Evaluation of flare-ups* below) confirmed by the Investigator, and randomized to study medication within 7 days of flare-up onset, were eligible to take part in the trial. Sexually active participants must have agreed to remain abstinent or use two highly effective forms of birth control [26].

#### PVO-1A-202 (open-label extension)

PVO-1A-202 (NCT02279095; identified as PVO-1A-204 [NCT02979769; registered 02/12/2016] in France [30]; **Supplementary Fig. 1C**) was an open-label extension of PVO-1A-201, which further evaluated the efficacy and safety of various palovarotene treatment regimens. The trial was conducted between October 2014 and September 2022 and was divided into Parts A, B, C, and D. The key flare-up outcome was the incidence and volume of new HO at Week 12 as assessed by flare-up body region CT scan. The primary disease progression endpoint was the annualized change in new HO volume as assessed by low-dose WBCT every 12 months up to 72 months, assessed only in participants who received chronic treatment with palovarotene in PVO-‍1A-‍202 Part B or Part C.

In Part A, palovarotene 10/5 mg flare-up treatment was administered for 6 weeks followed by a 6-week follow-up period in participants reporting a qualifying flare-up (see *Evaluation of Flare-Ups* below).

In Part B, palovarotene was administered to all participants when qualifying flare-ups (see *Evaluation of Flare-Ups* below) occurred, dosed 20 mg (high-dose palovarotene treatment) for 4 weeks followed by 10 mg (low-dose palovarotene treatment) for 8 weeks (palovarotene 20/10 mg flare-up treatment; 10 mg palovarotene treatment could be extended in 4-week intervals until flare-up resolution). Palovarotene treatment was weight-adjusted for skeletally immature participants (< 18 years of age and < 90% skeletal maturity on hand/wrist radiography [defined by a bone age of < 12 years in female participants and < 14 years in male participants]). In addition, palovarotene 5 mg chronic treatment was administered daily for up to 24 months in skeletally mature participants (≥ 18 years of age or ≥ 90% skeletal maturity on hand/wrist radiography [defined by a bone age of ≥ 12 years in female participants and ≥ 14 years in male participants]).

In Part C, all participants received chronic treatment (5 mg daily for up to 36 months; weight-adjusted) and the 20/10 mg flare-up treatment (weight-adjusted) was administered to all participants with a qualifying flare-up (see *Evaluation of Flare-Ups* below) or Investigator-confirmed high-risk traumatic event. Participants who experienced a new intercurrent flare-up (defined as a new flare-up or a marked worsening of the original flare-up) or had an Investigator-confirmed high-risk traumatic event during flare-up treatment restarted 20/10 mg flare-up treatment (i.e., 20 mg for 4 weeks followed by 10 mg for 8 weeks; weight-adjusted).

In Part D, annual assessments were obtained following the last dose of palovarotene in participants who were skeletally immature at the time of treatment discontinuation to obtain longer-term safety data. The duration of Part C and Part D together was limited to 48 months.

Individuals with clinically diagnosed FOP with the *ALK2/ACVR1*^R206H^ mutation who were enrolled in PVO-1A-201 were eligible to take part in PVO-1A-202 Parts A and B; 18 new participants were also enrolled in Part B. All participants enrolled in Part B were eligible to participate in Part C, and no new participants were enrolled in Part C and D.

Participants in all studies were required to provide written, informed consent, and studies were approved by independent ethics committees or institutional review boards. Participants were able to progress from early studies onto later studies, as displayed in Fig. 1. Notably, participants could contribute to more than one flare-up outcome across the program. For example, a participant could have an untreated flare-up in the NHS and a palovarotene-treated flare-up in PVO-1A-201 and/or PVO-1A-202.

### Study outcomes

Details of all primary and secondary study endpoints are listed in Table 1. Flare-up outcomes were generally measured at Baseline (defined as Flare-Up Day 1) and Week 12 (defined as Flare-Up Day 84). Disease progression endpoints were generally measured annually. The following outcomes were assessed in all three studies, unless specified otherwise.

**Table 1:** The suitability of endpoints included in PVO-1A-001, PVO-1A-201, and PVO-1A-202

**PVO-1A-001 (NHS)**			
**Key endpoints**	**Secondary endpoints** **(12-week flare-up progression)**	**Secondary endpoints** **(disease progression)**	**Suitability of endpoints**
Flare-up outcomes: Incidence and volume of new HO at Week 12 as assessed by flare-up body region CT scan Disease progression: Change from Baseline in the total body burden of HO as assessed annually by low-dose WBCT (excluding the head) over 36 months	• Pain and swelling at the flare-up body region using numeric rating scale for each symptom • Biomarkers^a^ • Physical function as assessed by ROM (goniometer) • Disease-specific patient-reported outcome measure (FOP-PFQ) • Patient-reported outcome measure of physical and mental health (PROMIS Global Health Scale)	• ROM as assessed by CAJIS for FOP • Patient-reported use of aids, assistive devices, and adaptations • Disease-specific patient-reported outcome measure (FOP-PFQ) • Patient-reported measure of physical and mental health (PROMIS Global Health Scale) • Biomarkers	• Comparison of DXA and low-dose WBCT (excluding the head) determined that low-dose WBCT was the preferred imaging modality for measuring HO [31]. When measured by low-dose WBCT, total HO volume increased substantially over the course of the study, suggesting the usefulness of this approach as a meaningful endpoint to measure disease progression [24] • Use of aids, assistive devices, and adaptations also increased substantially across the study, suggesting that this endpoint may provide a valuable real-world indicator of decreased mobility [24] • Functional and patient-reported outcome assessments, such as CAJIS and FOP-PFQ, showed limited changes during the course of the study. A longer study duration would be required to detect substantial changes in these endpoints [24]
**PVO-1A-201 (Phase II RCT)**			
**Key endpoints**	**Secondary endpoints** **(12-week flare-up progression)**	**Secondary endpoints** **(disease progression)**	**Suitability of endpoints**
Flare-up outcomes: Incidence and volume of new HO at Week 12, assessed by flare-up body region CT scan Disease progression: None	• Presence of soft tissue edema and/or cartilage as assessed by MRI (or soft tissue edema by ultrasound in participants unable to undergo MRI) • Active ROM measured by goniometer at flare-up body region • Patient and Investigator global assessment of movement • Pain and swelling at the flare-up body region using a numeric rating scale for each symptom • Use of aids, assistive devices, and adaptations for daily living • Disease-specific patient-reported outcome measure (FOP-PFQ) • Biomarkers^a^ • Steady-state pharmacokinetics • Safety evaluation, including: adverse events, clinical safety laboratory parameters, vital signs, concomitant medications, assessment of suicide ideation/behavior using the C-SSRS, and assessment of physeal growth plate and linear growth in participants under the age of 18 years	• None	• Plain radiographs were insufficiently sensitive in identifying or quantifying new HO. Therefore, CT scans were considered the preferred imaging modality [26]; however, plain radiographs remained the most useful imaging approach for growth plate assessments • The 12-week period over which the trial was conducted may have been too short for new HO to be fully observed [26] • More than half of all patient-reported flare-ups did not result in new HO at the associated body region. This suggests that there may have been abortive flare-ups, or that symptoms were related to other disease processes [26]
**PVO-1A-202 (Phase II OLE)**			
**Key endpoints**	**Secondary endpoints (12-week flare-up progression)**	**Secondary endpoints (disease progression)**	**Suitability of endpoints**
Flare-up outcomes: Incidence and volume of new HO at Week 12 as assessed by flare-up body region CT scan Disease progression: Change from Baseline in the total body burden of HO as assessed by low-dose WBCT (excluding the head) up to Month 72	• Presence of soft tissue edema and/or cartilage as assessed by MRI (or soft tissue edema by ultrasound in participants unable to undergo MRI) • Active ROM measured by goniometer at flare-up body region • Patient and Investigator global assessment of movement • Pain and swelling at the flare-up body region using a numeric rating scale for each symptom • Use of aids, assistive devices, and adaptations for daily living • Disease-specific patient-reported outcome measure (FOP-PFQ) • Biomarkers^a^ • Steady-state pharmacokinetics • Safety evaluation, including: adverse events, clinical safety laboratory parameters, vital signs, concomitant medications, assessment of suicide ideation/behavior using the C-SSRS, and assessment of physeal growth plate and linear growth in participants under the age of 18 years	• The proportion of participants with any new HO • ROM as assessed by CAJIS for FOP • Disease-specific patient-reported outcome measure (FOP-PFQ) • Patient-reported measure of physical and mental health (PROMIS Global Health Scale)	• Not applicable as trial results are not yet publicly available in a peer-reviewed publication

^a^Osteocalcin, bone-specific alkaline phosphatase, P1CP-C-terminal propeptide of type 1 procollagen, P1NP-N-terminal propeptide of type 1 procollagen, cartilage-derived retinoic acid-sensitive protein, C-terminal telopeptide, urinary basic fibroblast growth factor, erythrocyte sedimentation rate, C-reactive protein, interleukin-6, interleukin-1 beta, tumor necrosis factor-alpha, creatine phosphokinase, and lactate dehydrogenase.

CAJIS: Cumulative Analogue Joint Involvement Scale; C-SSRS: Columbia-Suicide Severity Rating Scale; CT: computed tomography; DXA: dual-energy X-ray absorptiometry; FOP: fibrodysplasia ossificans progressiva; FOP-PFQ: FOP physical function questionnaire; HO: heterotopic ossification; MRI: magnetic resonance imaging; NHS: natural history study; OLE: open-label extension; PROMIS: Patient-Reported Outcomes Measurement Information System; RCT: randomized controlled trial; ROM: range of motion; WBCT: whole-body CT.

#### Evaluation of flare-ups

Patient-reported flare-up endpoints were generally evaluated at Baseline and Week 12. Flare-ups were reported by participants and, to provide an element of certainty in the occurrence of a flare-up based on prior knowledge, confirmed by an Investigator according to the presence of the following symptoms: pain, swelling, stiffness, decreased range of motion (ROM), redness, and warmth [8]. Qualifying flare-ups were defined by ≥ 2 of the aforementioned symptoms in PVO-‍1A-‍‍201 and PVO-‍1A-‍202 Parts A and B, and ≥ 1 symptom in PVO-‍1A-‍202 Part C. Flare-up symptoms were required to be consistent with a participant’s previous flare-up, with treatment initiated within 7 days of flareup onset (or imaging performed within 14 days of flare-up onset in the NHS; only one flare-up per year was allowed to be evaluated in clinic in the NHS) to qualify for evaluation. Details of flare-up body region, probable causes, duration, number and type of symptoms, presence of pain and/or swelling, and whether the participant received steroid treatment for the flareup, were recorded. Flare-up body regions were assessed for the presence of soft tissue edema using magnetic resonance imaging (MRI), or musculoskeletal ultrasound in participants who were unable to undergo MRI.

#### Imaging of heterotopic ossification

For flare-up outcomes, flare-up body regions were imaged over the course of the 12-week flare-up treatment and assessment period (at Baseline, Week 6, and Week 12), to determine the incidence and volume of new HO. In the initial study designs across the NHS, PVO-1A-201, and PVO-1A-202 Part A, incidence of new HO was assessed by plain radiographs and low dose CT scan at the flare-up body region. Once it was determined that plain radiographs were less sensitive than CT scans [25, 26], and to minimize radiation exposure, all subsequent flare-up HO imaging was performed by CT at Week 12 only across all studies. For disease progression outcomes, low-dose WBCT (excluding the head) was obtained annually for participants in the NHS and PVO-1A-202 Parts B and C to assess incidence and volume of new HO [24]. All imaging (both flare-up body region and whole body) was performed using predefined procedures and interpreted in a blinded manner by trained radiologists in a central imaging facility.

### Clinical outcomes

For flare-up outcomes, ROM was assessed at Week 12 using the 5-point participant and Investigator global assessments of movement scale. Scores assessed range from “better movement compared with Flare-Up Day 1” to “severely worse movement compared with Flare-Up Day 1”. Active ROM was also assessed at the flare-up joint using a goniometer by qualified site personnel, with the percentage of normal total arc of motion determined.

For disease progression outcomes in the NHS and PVO-1A-202, ROM was assessed annually using the CAJIS for FOP [28], a scale assessed by the Investigator measuring ROM across 12 joints (shoulder, elbow, wrist, hip, knee, and ankle on both right and left sides), and three body regions (jaw, cervical spine [neck], and thoraco-lumbar spine) [24]. Each joint or region was assigned a score (0 = uninvolved; 1 = partially involved; 2 = completely ankylosed), with a maximum total score of 30 [28]. The CAJIS for FOP also included an assessment of activities of daily living and ambulation.

#### Patient reported outcomes

Patient-reported physical function was assessed over the course of flare-ups and annually using the FOP Physical Function Questionnaire (FOP-PFQ), which was designed specifically for use in individuals with FOP [32, 33]. This disease-specific questionnaire assesses functional impairment through questions about activities of daily living and physical functioning. Age-appropriate forms were self-completed by adults (participants ≥ 15 years), with the option for self-completion (for participants 8–14 years) or completion by a proxy (for participants ≤ 14 years); specific instructions were provided to participants and proxies. Analyses were performed on transformed scores, expressed as a percentage of the worst possible score (0–100%; lower scores indicate worse physical function).

Overall physical and mental health was assessed over the course of flare-ups and annually using age-specific assessments: the Patient-Reported Outcome Measure Information System (PROMIS) Global Physical and Mental Health Scale for participants ≥ 15 years, and the PROMIS Pediatric Global Health Scale for participants ≤ 14 years, with the option for self-completion (for participants 8–14 years) or completion by a proxy (for participants ≤ 14 years). These scales measure what individuals are able to do and how they feel [34, 35]. As with the FOP-PFQ, specific instructions were provided to participants and proxies. Scores were converted to T-scores, such that a value of 50 (with a standard deviation of 10) represents the average for the general population in the United States. The use of aids, assistive devices, and adaptations was assessed longitudinally in the NHS and PVO-1A-202 Part B, and over the course of flare-ups in PVO-1A-201 and PVO-1A-202 Part A [24].

#### Exploratory outcomes

Blood and urine samples were collected from participants for assessment of biomarkers of cartilage and bone (osteocalcin, bone-specific alkaline phosphatase, C-terminal propeptide of type 1 procollagen, N-terminal propeptide of type 1 procollagen, collagen-derived retinoic acid protein, and C-terminal telopeptide), angiogenesis (ratio of urinary fibroblast growth factor and urine creatine) and inflammation (erythrocyte sedimentation rate, C-reactive protein, interleukin-6, interleukin-1 beta, tumor necrosis factor-alpha, creatine phosphokinase, and lactate dehydrogenase). In the NHS, biomarkers were assessed at Baseline and Months 12, 24, and 36, and at Baseline, Week 6, and Week 12 for imaged flare-ups. In PVO-1A-201 and in PVO-1A-202 Part A, biomarkers were assessed at Baseline and Weeks 2, 4, 6, and 12 during flare-ups and also at Month 12 in PVO-1A-202 Part A [26]. In PVO-1A-202 Part B, biomarkers were assessed at Months 12 and 24, and at Baseline and Weeks 4, 8, and 12 in participants experiencing a flare-up. Based on emerging data, biomarkers were removed from the evaluation for PVO-1A-202 Part C and the later stages of the NHS (October 2017 onwards).

### Safety

Safety was monitored throughout all studies, including the NHS [24, 26]. Adverse events (AEs; assessed as mild, moderate, or severe) were recorded and coded using the Medical Dictionary for Regulatory Activities (MedDRA, version 17.0). While no pharmacological intervention was applied in the NHS, AEs resulting from any protocol-specified procedure were recorded [24]. In addition, medical events (using a standardized checklist) were collected at Baseline and annually in the NHS [24].

Other safety evaluations included a physical examination, assessment of suicide ideation/behavior using the Columbia-Suicide Severity Rating Scale (C-SSRS; based on the established safety profile of other oral systemic retinoids) [36], laboratory parameters (hematology, biochemistry, and urinalysis), electrocardiograms, body weight, vital signs, urine pregnancy tests, and concomitant medications. In the NHS, pulmonary function and pulse oximetry were also assessed [24]. Participants aged ≤ 18 years with open epiphyseal plates had these evaluated using knee (anterior/posterior view) and hand/wrist (posterior/anterior view) radiographs in the phase II trials and the later stages of the NHS (October 2017 onwards). Linear growth was assessed by height using a stadiometer and knee height (in triplicate) in the NHS and phase II trials. Femur and tibia length and bilateral hand/wrist and knee growth plates were assessed by lowdose WBCT scan in the NHS and PVO-1A-202 Parts B and C.

### Statistical analyses and sample sizes

As a general overview of the statistical approaches utilized across these studies, descriptive statistics were used for continuous data and included the number, mean, standard deviation, standard error, median, minimum, and maximum. Categorical data were summarized using counts and percentages. For PVO-1A-201, the primary efficacy analysis was assessed using the Cochran-Armitage test of trend (one-sided), to assess the overall trend of response across the treatment groups, and continuously scaled parameters were analyzed using repeated measures mixed models [26]. To distinguish flare-up-based analyses from participant-based analyses across all studies, m was used for number of flare-ups and n was used for number of participants. Further details of specific analyses for individual study outcomes are provided in the publications reporting study results for the NHS [24] and PVO-1A-201 [26], and will be publicly available for PVO-1A-202 in due course [27].

For the NHS, a sample size of up to 100 participants was selected, which was judged to provide sufficient precision for point estimates of measured parameters [24]. For PVO-1A-201, 21 participants were enrolled to the palovarotene 10/5 mg flare-up treatment group, 9 participants were enrolled to the palovarotene 5/2.5 mg flare-up treatment group, and 10 participants were enrolled to the placebo group [26]. This sample size was determined based on retrospective data estimating that 20% of flare-ups in untreated individuals would result in no or minimal HO (estimated using percentage of flare-ups with no change in movement or function at the flare-up body region) [8], and the hypothesis that 80% of flare-ups in palovarotene-treated individuals will result in no or minimal new HO based on animal models [37]. The sample size was based on these assumptions and on the desire to detect a linear trend over the dose range. For PVO-1A-202 Part A, the sample size was based on the number of participants who had completed PVO-1A-201 (up to 40 adult and pediatric participants) [26]. For PVO-1A-202 Part B, the sample size was up to 60 participants to allow for the collection of data on the targeted number of overall flare-ups. No new participants were enrolled in PVO-1A-202 Part C or D.

### Protocol amendments

FOP is an ultra-rare disease in which a limited number of clinical trials have been conducted. Therefore, the palovarotene clinical development program was designed to allow for modifications to the individual trial designs between PVO-1A-201 (the randomized placebo-controlled trial) and PVO-1A-202 (the open-label extension), and between the parts of PVO-1A-202.

## Results

In total, 114 individuals with FOP were enrolled in the NHS and 58 individuals with FOP were enrolled in the phase II trials. Results for the NHS and PVO-1A-201 have been reported [24, 26], and complete results for PVO-1A-202 will be publicly available in due course.

Results of the NHS comprehensively documented FOP disease progression over three years, confirming that HO in individuals with FOP is severely debilitating and is associated with restriction of movement and cumulative disability. Mean annualized new HO volume was highest for participants aged 8 to < 15 years and 15 to < 25 years, and lowest for those aged 25–65 years. Over the course of the NHS, 90.2% of participants began using a new aid, assistive device, or adaptation [24].

When palovarotene was administered for 6 weeks at the onset of a flare-up in PVO-1A-201, a numerically lower proportion of participants experienced new HO and had lower volume of new HO at the flare-up region compared with placebo. Palovarotene was well tolerated and the safety profile was similar to other retinoids. Although no statistically significant trend associated with palovarotene treatment was identified in PVO-1A-201, the findings supported further evaluation of palovarotene for the reduction of new HO in larger populations of individuals with FOP [26].

The suitability of endpoints used in the NHS, PVO-1A-201, and PVO-1A-202 are outlined in Table 1 and the implications of these are discussed in more detail below.

## Discussion

**Fig. 2:**
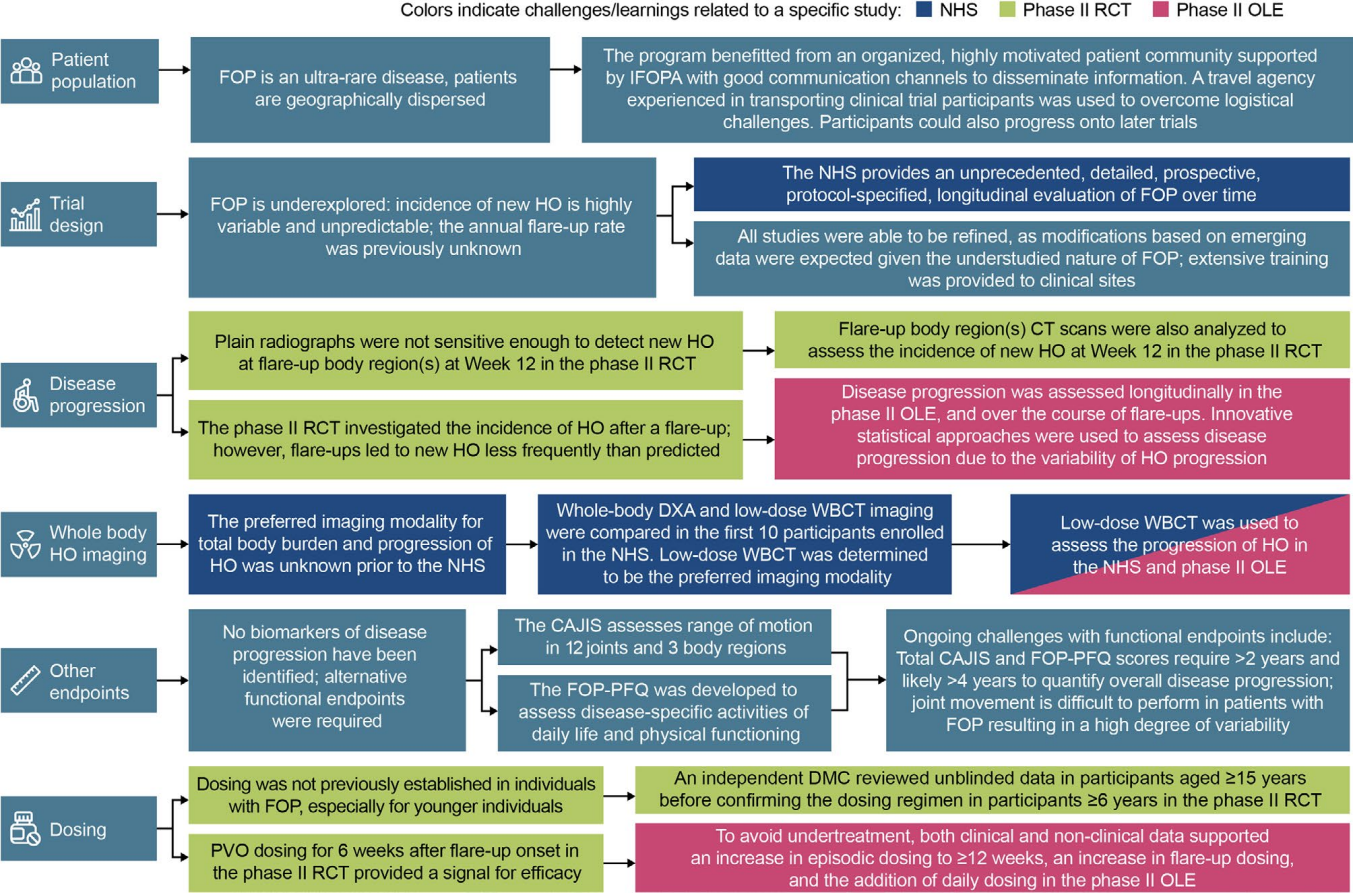
Key challenges and protocol amendments during the PVO-1A-001, PVO-1A-201, and PVO-1A-202 studies. CAJIS: Cumulative Analogue Joint Involvement Scale; CT: computed tomography; DMC: data monitoring committee; DXA: dual-energy X-ray absorptiometry; FOP: fibrodysplasia ossificans progressiva; FOP-PFQ: FOP Physical Function Questionnaire; HO: heterotopic ossification; IFOPA: International FOP Association; NHS: natural history study; OLE: open-label extension; PVO: palovarotene; RCT: randomized controlled trial; ROM: range of motion; WBCT: whole-body computed tomography

The NHS and phase II trials described here were developed to determine the natural history of FOP, both for disease progression and for flare-ups, to evaluate potential endpoints for utilization in future trials, to understand the efficacy and safety of palovarotene in individuals with FOP, and to establish appropriate palovarotene treatment regimens [24, 26, 27]. In order to develop treatments for ultra-rare diseases such as FOP, flexible methodological approaches and refinements to study designs are required to act on new knowledge and overcome the evolving challenges faced (Fig. 2). The palovarotene clinical development program for FOP revealed lessons that could inform future research, including the suitability of specific endpoints (Table 1) and how the way in which a study is designed can impact trial results, as discussed further below.

### Key challenges and protocol modifications

FOP, like most ultra-rare diseases, is underexplored, with highly variable and unpredictable disease progression, flare-up symptoms and outcomes (i.e., the presence of new HO), variable comorbidities, and a lack of robust biomarkers [8, 11]. Therefore, at the outset of this clinical development program, a detailed, longitudinal evaluation of FOP was undertaken through the NHS [24]. This included numerous possible endpoints for interventional trials, such as evaluation of flare-up outcomes, annual evaluation of disease progression, and functional and patient-reported outcomes. Furthermore, all studies were able to be quickly refined based on emerging nonclinical data, including studies in animal models, and clinical data, including from the NHS and unblinded PVO-‍1A-‍202 trial. This allowed for the refinement of protocols while studies were ongoing and as learnings evolved to ensure an optimal approach, as the robustness of the endpoints was not known due to the understudied nature of FOP. Protocol amendments were made for various reasons, including limiting the burden of participation, for example by the removal of biomarker evaluation from PVO-‍1A-‍202 Part C and the later stages of the NHS, and improving participant safety, for example by the minimization of radiation exposure through the use of low-dose WBCT scans, changing the indications for imaging, and using only CT scans to assess new HO volume [26]. Amendments were also made to optimize the palovarotene treatment regimen and to increase the understanding of FOP outcome progression.

Although palovarotene had previously been evaluated in clinical trials in chronic obstructive pulmonary disorder (COPD) [38, 39], the most appropriate treatment regimen for individuals with FOP with an active flare-up was yet to be established prior to this clinical development program. To address this, a data monitoring committee reviewed unblinded, preliminary efficacy and safety data in participants in the first cohort enrolled in PVO-1A-201, aged ≥ 15 years, prior to the enrolment of the second cohort of participants, aged ≥ 6 years [26]. The treatment regimen was further refined for PVO-1A-202, in which flare-up treatment was combined with chronic treatment, following evidence that this prevented HO in an animal model of FOP [17].

Another key challenge associated with this clinical development program was that, as FOP is an ultra-rare disease, few individuals with FOP are in the same geographic locality as the research sites. This required careful consideration when designing study methodology and determining operational logistics to ensure sufficient numbers of enrolled participants, and was particularly important for those studies that enrolled participants at the time of a flare-up. The presence of an organized and highly motivated international patient community, with good communication channels, benefitted the program and enabled the effective dissemination of information to individuals with FOP. Additionally, a travel agency experienced in transporting clinical trial participants and conducting remote visits were used to overcome the geographical and substantial physical challenges faced by participants. Participants could also progress from early studies into later studies, if desired. Logistical challenges were further exacerbated by the onset of the COVID-19 pandemic, which prompted a switch from onsite to remote assessments via telemedicine; participant retention and the quality of data collection were key considerations when determining which assessments could be conducted remotely.

### Suitability of endpoints

Trial endpoints in clinical development programs must be clinically meaningful to determine whether an investigational therapeutic offers a benefit for patients [32]. However, the understudied nature of FOP presented a challenge in the selection of meaningful study endpoints.

Prior to the NHS, the optimum imaging modality for assessing the total body burden of HO and HO progression was unknown. Both whole-body dual-energy X-ray absorptiometry (DXA) and low-dose WBCT imaging were compared in the first 10 participants enrolled in the NHS, and it was determined that low-dose WBCT was the preferred imaging modality [31]. Therefore, this imaging approach was used to assess the progression of HO in the remaining participants in the NHS and for all participants in PVO-1A-202. Additionally, in the initial study designs of the NHS, PVO-1A-201, and PVO-1A-202 Part A, plain radiographs were used alongside low-dose CT to determine the incidence and volume of new HO during flare-ups; however, it was subsequently determined that plain radiographs were not sensitive enough to detect new HO at Week 12 [26]. Therefore, the protocols were amended such that all subsequent flare-up HO imaging was performed by low-dose CT only across all studies.

The sensitivity of patient-reported and physical function measures in detecting FOP disease progression was unknown before this clinical development program was established, as was the correlation of total body burden of HO with these measures. Furthermore, no clinically reliable biomarkers of disease progression or flare-ups have been identified in FOP [11]. Therefore, alternative disease-specific patient-reported and functional endpoints were included to assess the impact of HO on patient wellbeing and quality of life. The CAJIS was developed independently and used to globally and rapidly assess overall joint involvement and ROM [28]. The FOP-PFQ was developed specifically for this program, as there was no existing way to assess disease-specific activities of daily living and physical functioning in individuals with FOP [32, 33]. However, there are ongoing challenges associated with these measures, as it can be difficult to evaluate disease progression over the relatively short time period of a clinical trial [32]. Based on accumulating data, average CAJIS total scores increase by approximately 0.5 points per year in individuals with FOP (out of a total of 30), meaning that a period of approximately 4 years would be required for a 2-point change in total score, which equates to complete ankylosis of a single joint or partial restriction of several joints [28, 32]. Accordingly, results from the NHS showed limited changes in CAJIS and FOP-PFQ over the 3-year study duration, and indicate that a longer duration would be required to detect substantial changes in these endpoints [24]. In contrast, the use of aids, assistive devices, and adaptations increased substantially over the course of the NHS, suggesting that this endpoint may provide a valuable real-world indicator of decreased mobility [24].

### Impact of study design on results of the NHS and PVO-1A-201

Designing clinical trials requires careful consideration, as decisions regarding trial design can have important impacts on study results. The NHS described here incorporated numerous endpoints, including functional, patient-reported, and laboratory outcomes, in order to determine which ones would be optimal to measure disease progression over time. Including a variety of endpoints allowed for a cross-sectional analysis of Baseline data by participant age [32], and subsequently a prospective analysis of endpoints over time, once sufficient follow-up data was available [24]. Because of this, the design of the NHS enabled the ongoing assessment of the suitability of endpoints for future interventional trials.

PVO-1A-201 was the first interventional study in the clinical program, and therefore the most appropriate treatment regimen for individuals with FOP was not known at trial commencement. This limited prior knowledge impacted the study results, with no statistically significant trend associated with palovarotene treatment being identified [26]. Subsequently, data from PVO-1A-201, PVO-1A-202 Part A, and non-clinical studies led to the palovarotene treatment regimen being amended for later trials to treat flare-ups for 12 weeks at a higher dose.

### Impact of the NHS and phase II trials on the phase III MOVE trial

Lessons learned from the NHS and phase II trials described here informed the design of the phase III MOVE trial (PVO-1A-301 [NCT03312634; registered 18/10/2017]), including the palovarotene treatment regimen that was evaluated, the implementation of palovarotene treatment for all flare-ups, and the endpoints that were measured [40, 41].

MOVE was a single-arm, open-label trial in which participants received palovarotene 5 mg chronic treatment once daily alongside palovarotene 20/10 mg flare-up treatment (weight-adjusted in skeletally immature patients) at the time of flare-ups, in order to further assess efficacy and safety of palovarotene in patients with FOP. Data from untreated individuals enrolled in the NHS, who were representative of the world-wide population of patients with FOP, provided the comparator arm for the MOVE trial, with the primary endpoint of annualized change in new HO volume assessed by low-dose WBCT [41]. Interim post hoc analyses of MOVE showed substantial efficacy of palovarotene, as measured by reduction in new HO volume compared with participants in the NHS. As expected, due to the short treatment duration in the interim analysis, no substantial changes in functional outcomes and quality of life were observed. Palovarotene was generally well tolerated, with the notable exception of the risk of premature physeal closure (PPC) in skeletally immature participants [41].

### Ongoing and future work in FOP

Other potential disease-modifying treatments, with a range of mechanisms of action, are currently under investigation for FOP, including garetosmab (REGN2477; NCT03188666 [LUMINA-1]) [42], fidrisertib (IPN60130; previously known as BLU-782; NCT05039515 [FALKON]) [43], saracatinib (NCT04307953 [STOPFOP]) [44], rapamycin [45], and INCB000928 (NCT05090891 [PROGRESS]) [46]. The planned phase II trials of IPN60130 and other ALK2 inhibitors, and the ongoing phase II trial of saracatinib, in patients with FOP will also determine efficacy by using imaging to assess volumetric accumulation of HO.

## Conclusions

Across the palovarotene clinical development program, including the phase II trials and MOVE, 164 individuals with FOP received multiple doses of palovarotene. The studies described here, including the treatment regimens, were modified based on emerging non-clinical and clinical data, allowing timely refinement of protocols while studies were ongoing. Many of the lessons learned throughout the numerous iterations of the palovarotene clinical development program, including the suitability of specific endpoints and the implications of study design on trial results, have been incorporated into the guidelines for clinical trials in FOP [47]. This important narrative could be used as an example to inform the design of future clinical trials.

### Electronic supplementary material

Below is the link to the electronic supplementary material.

Supplementary Material 1

## Data Availability

Qualified researchers may request access to patient-level study data that underlie the results reported in this publication. Additional relevant study documents, including the clinical study report, study protocol with any amendments, annotated case report form, statistical analysis plan and dataset specifications may also be made available. Patient-level data will be anonymized, and study documents will be redacted to protect the privacy of study participants. Where applicable, data from eligible studies are available 6 months after the studied medicine and indication have been approved in the US and EU or after the primary manuscript describing the results has been accepted for publication, whichever is later. Further details on Ipsen’s sharing criteria, eligible studies and process for sharing are available here (https://vivli.org/members/ourmembers/). Any requests should be submitted to www.vivli.org for assessment by an independent scientific review board.

## Declarations

The IRBs/IECs that reviewed and approved the study documents for PVO-1A-001 were: University of Pennsylvania Office of Regulatory Affairs, US; Human Research Protection Program Institutional Review Board, University of California San Francisco, US; Comité de Protection des Personnes Île de France II, Hôpital Necker Enfants Malades, France; Comitato Etico Regionale, IRCCS AOU San Martino IST, Italy; Comite de Etica de Protocolos de Investigacion, Hospital Italiano de Buenos Aires, Argentina; London – Stanmore Research Ethics Committee, Health Research Authority, UK; and Metro South Human Research Ethics Committee, Australia.

The IRBs/IECs that reviewed and approved the trial documents for PVO-1A-201 were: Ethics Committee Île de France VIII, France; Human Research Protection Program Committee on Human Research, University of California, San Francisco, US; Institutional Review Board, University of Pennsylvania, US; and NRES Committee South East Coast – Brighton & Sussex, UK.

The IRBs/IECs that reviewed and approved the study documents for PVO-1A-202 were: University of Pennsylvania Institutional Review Board, US; University of California San Francisco Human Research Protection Program Institutional Review Board, US; Mayo Clinic Institutional Review Boards, US; Comité de Protection des Personnes Île de France VIII, Hôpital Ambroise Paré, France; NHS Health Research Authority London – Brighton & Sussex Research Ethics Committee, UK; Comité de Ética de Protocolos de Investigacion, Argentina; and Metro South Human Research Ethics Committee, Australia.

Prior to participation in the studies, the Investigator and/or delegate fully explained to the patients and/or parents all aspects of the study that were relevant to the decision to participate. Informed consent was sent to the patients and/or parents for reading and reflection, and was documented by means of a written, signed, and dated consent form, prior to study initiation.

## List of abbreviations

ACVR1: Activin A receptor type I
AE: adverse event
ALK2: Activin-like kinase 2
BMP: bone morphogenetic protein
C-SSRS: Columbia-Suicide Severity Rating Scale
CAJIS: Cumulative Analogue Joint Involvement Scale
CT: computed tomography
DMC: data monitoring committee
DXA: dual-energy X-ray absorptiometry
FOP: fibrodysplasia ossificans progressive
FOP-PFQ: FOP Physical Function Questionnaire
HO: heterotopic ossification
ICC: International Clinical Council
MedDRA: Medical Dictionary for Regulatory Activities
MRI: magnetic resonance imaging
NHS: natural history study
OLE: open-label extension
PROMIS: Patient-Reported Outcome Measure Information System
PVO: palovarotene
RARγ: retinoic acid receptor gamma
RCT: randomized controlled trial
ROM: range of motion
WBCT: whole-body computed tomography
