# Supplementary material for: Study methodology and insights from the palovarotene clinical development program in fibrodysplasia ossificans progressiva
Source: BMC Med Res Methodol. 2023 Nov 13;23:269. doi: 10.1186/s12874-023-02080-7 (PMC10642058; doi:10.1186/s12874-023-02080-7)
Supplement: Supplementary file 1 — Supplementary Material 1 [file 12874_2023_2080_MOESM1_ESM.pdf]

Supplementary Figure 1. Study designs of the PVO-1A-001, PVO-1A-201 and PVO-1A-202 studies

Supplementary Figure 1A. PVO-1A-001 natural history study

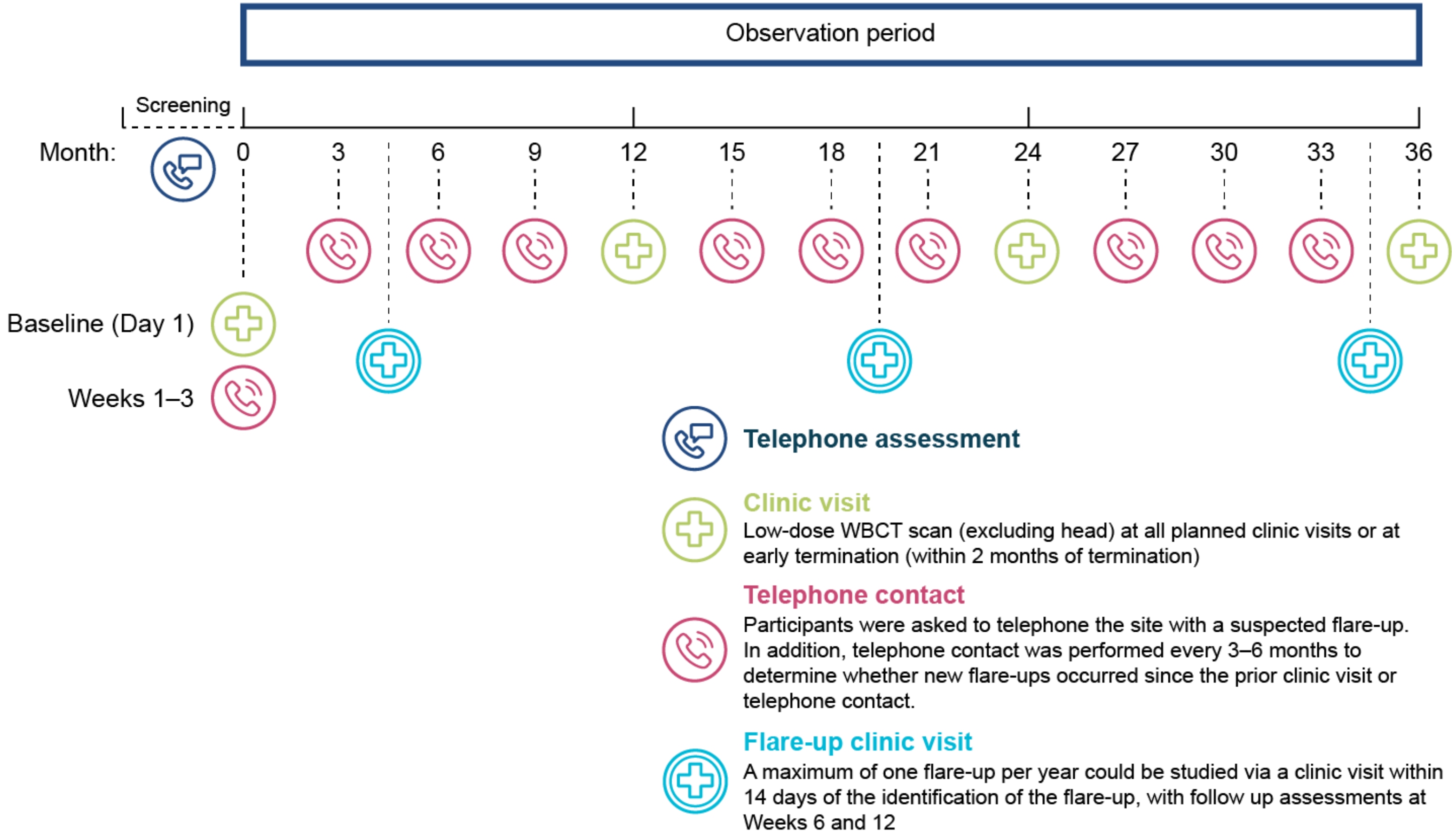

WBCT: whole-body computed tomography.

Supplementary Figure 1B. PVO-1A-201 randomized control trial

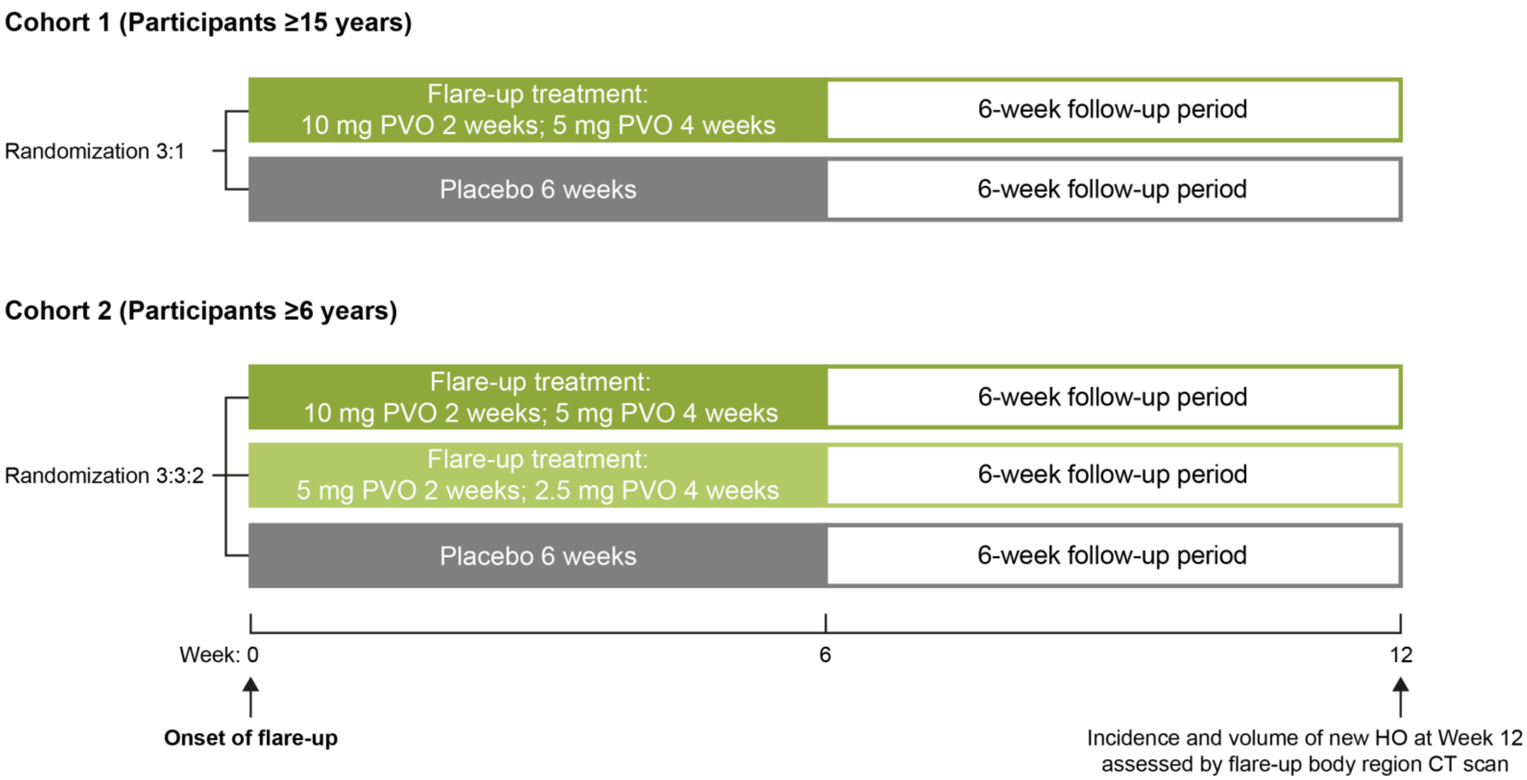

CT: computed tomography; HO: heterotopic ossification; PVO: palovarotene.

Supplementary Figure 1C. PVO-1A-202 open-label extension trial

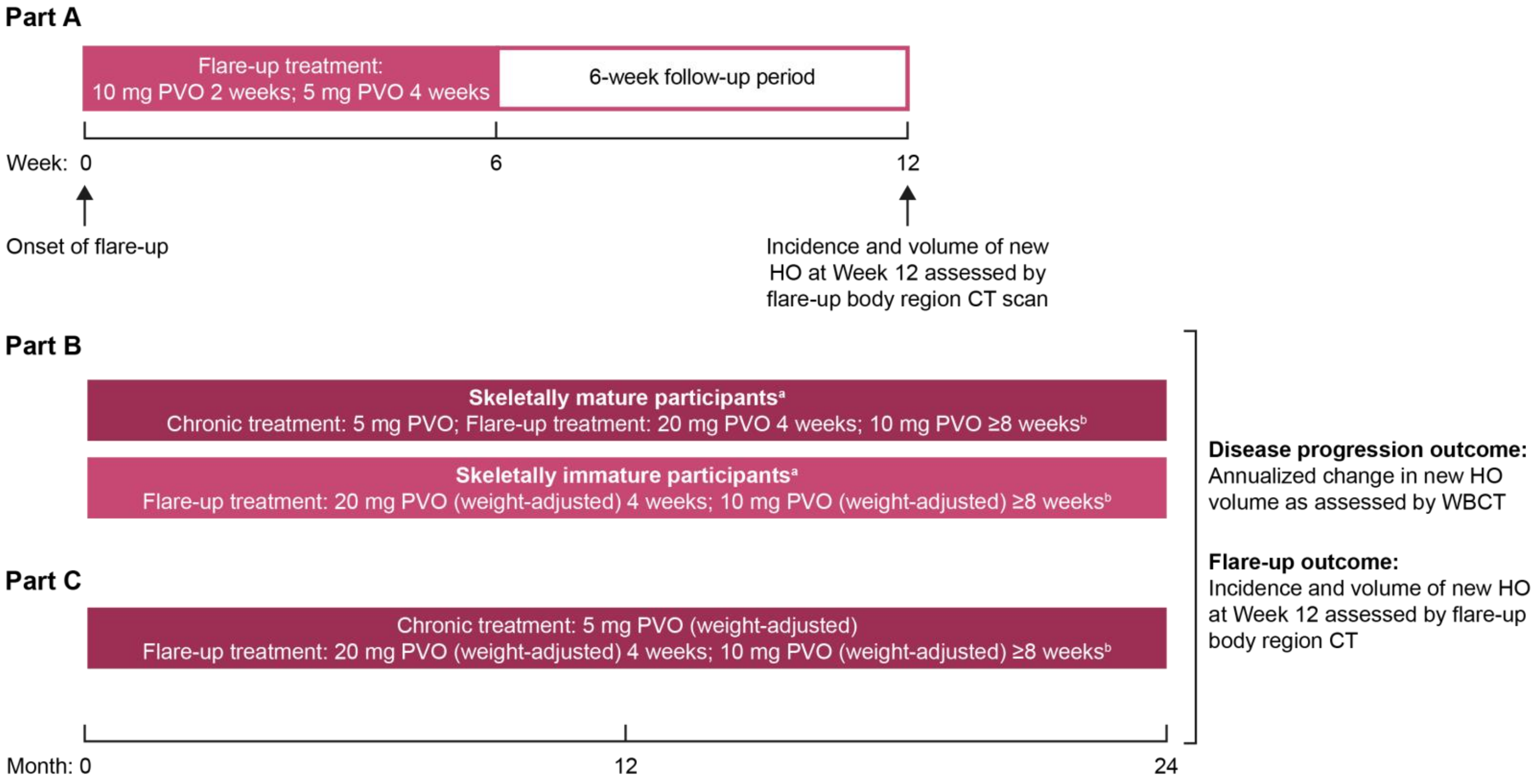

In Part D of PVO-1A-202, annual assessments were obtained after the last dose of palovarotene in participants who were skeletally immature at the time of treatment discontinuation to obtain longer-term safety data. [a]Skeletally mature participants were ≥18 years of age or had ≥90% skeletal maturity on hand/wrist radiography [defined by a bone age of ≥12 years in female and ≥14 years in male participants] at screening. Skeletally immature participants were <18 years of age and had <90% skeletal maturity on hand/wrist radiography at screening. [b]≥8 weeks or until flare-up resolved. CT: computed tomography; HO: heterotopic ossification; PVO: palovarotene; WBCT: whole-body computed tomography.
